# *TTBK2* ^T3290C^ mutation in spinocerebellar ataxia 11 interferes with ciliogenesis

**DOI:** 10.1515/tnsci-2022-0353

**Published:** 2024-10-03

**Authors:** Ruiqing Luo, Xiaoxia Zeng, Ping Li, Shuai Hu, Xueliang Qi

**Affiliations:** Department of Neurology, The Second Affiliated Hospital of Nanchang University, No. 1 Minde Road, Nanchang, Jiangxi, 330006, China

**Keywords:** SCA11, *TTBK2*, ciliogenesis, Cep164, missense mutation

## Abstract

This study aimed to elucidate the impact of the *TTBK2* ^T3290C^ mutation (MUT) associated with Spinocerebellar Ataxia 11 (SCA11) on TTBK2 expression, function, and ciliogenesis. Lymphocytes were isolated from peripheral blood samples of SCA11 family members with the MUT and healthy controls (wild-type, WT). HEK-293 cells transfected with either WT or MUT *TTBK2* plasmids were used to assess the MUT’s impact on TTBK2 protein expression, enzymatic activity, and its binding to Cep164 protein. Mouse embryonic fibroblast cells transfected with WT or MUT *TTBK2* plasmids examined the MUT’s effect on cilia formation. Clinically, there was no significant difference in the expression of TTBK2 between the SCA11 patients and healthy individuals. The *TTBK2* ^T3290C^ MUT did not affect protein expression or enzymatic activity but did reduce ciliary formation in embryonic cells and decreased binding affinity to Cep164. Therefore, our data suggested that the *TTBK2* ^T3290C^ MUT in SCA11 may impair ciliogenesis by weakening the interaction with Cep164.

## 1 Introduction

Spinocerebellar ataxia type 11 (SCA11) is an infrequent subtype of hereditary cerebellar ataxia exhibiting an autosomal dominant inheritance pattern. SCA11 is typified by a gradually progressive cerebellar ataxia, coupled with limb and gait ataxia, dysarthria, and oculomotor dysfunctions [1]. Globally, SCA11 is extremely rare, with only seven families reported to date [2]. The diagnosis of SCA11 predominantly relies on phenotypic presentation and the identification of pathogenic variants in the *TTBK2* gene, which remains the sole genetic locus linked to SCA11 [3,4].

The *TTBK2* gene, positioned on chromosome 15q15.2, encompasses 14 coding exons (with exon 1 being noncoding). It encodes a serine-threonine protein kinase, a member of the casein kinase 1 family [3]. The TTBK2 protein is ubiquitously expressed in adult tissues, predominantly in the brain and testes [5], where it demonstrates elevated expression and kinase activity. Notably, within the brain, TTBK2 is abundantly expressed across all regions, including the cerebellum, hippocampus, midbrain, and substantia nigra, albeit with lower levels in the cerebral cortex [3,6], underscoring its significance in neurological disorders.

Research indicates that mutations (MUTs) associated with SCA11 function as dominant negative alleles, producing a truncated protein (TTBK2^SCA11^) that disrupts the functionality of the full-length TTBK2 in ciliogenesis mediation [7]. Studies have elucidated the pivotal role of TTBK2 in initiating ciliogenesis, primarily through phosphorylation and/or protein interactions that govern the initiation, maintenance, stability, and transport of cilia [8,9,10]. Beyond its basal body localization, TTBK2 is also present at the ciliary transition zone, where it potentially regulates ciliogenesis homeostasis, thereby influencing ciliary length, stability, and intracellular transport. The findings of Bowie et al. [7] revealed that TTBK2 hypomorphic mutant cells exhibit reduced ciliary length and formation frequency, alongside perturbed trafficking of the Sonic Hedgehog pathway effector Smoothened and the structural regulator KIF7. Moreover, TTBK2 localizes to the maternal centriole, where it facilitates the removal of the negative regulatory factor (centriole curl protein 110) and the recruitment of positive regulatory factors and cargo carriers in cilia (intrabundle transport proteins 88, 140, and 81) [7,11]. This maternal centriole localization, mediated by Cep164, is crucial for ciliation initiation [12,13,14].

Our preliminary research identified a new heterozygous *TTBK2* MUT, c.3290T>C (exon 15), which led to an amino acid exchange (p.Val1097Ala) [15]. Therefore, this study was based on previous research to explore the effects of *TTBK2* MUT (c.3290T>C) on TTBK2 protein expression level, kinase activity, and interaction with Cep164 protein. By verifying the effect of *TTBK2* MUT on ciliary formation, the molecular mechanism of TTBK2 heterozygous MUT-induced SCA11 is revealed, bringing hope for the treatment of SCA11. Furthermore, enhancing awareness of SCA11 onset is advantageous for early diagnosis and the selection of appropriate treatment strategies, ultimately improving patient’s quality of life.

## 2 Materials and methods

### 2.1 Sample collection

All subjects originated from the same Chinese family afflicted with SCA11, as depicted in Figure S1. The family spanned three generations and comprised a total of 26 individuals, 10 of whom had manifested the disease across these generations. Clinical data were obtained from the 10 affected family members (I2, II2, II3, II5, II7, III3, III6, III8, III9, and III15). Following the acquisition of informed consent, 4 ml of venous blood was collected from the proband (III3) as well as from other affected family members (III6, III8, III9, and III15, carrying the pathogenic variant) and unaffected individuals (III4, III5, III7, III10, III12, and III16, wild type). The blood specimens were placed in ethylenediamine tetraacetic acid-containing anticoagulant tubes for lymphocyte isolation and subsequent lymphocyte separation for further analysis.

### 2.2 Reverse transcription-polymerase chain reaction (RT-PCR) analysis

Total RNA was extracted from isolated lymphocytes or transfected HEK-293/mouse embryonic fibroblast (MEF) cells using TRIzol reagent. Subsequently, cDNA synthesis was performed using the 5× Prime Script RT Master Mix, followed by real-time quantitative polymerase chain reaction with the SYBR Premix Ex Taq State II kit. The primer sequence is as follows: *TTBK2*(F): 5′CTCCTCACAATCCAAAAACACC3′, *TTBK2*(R): 5′CTAGATGGTGAGGAACTAGACG3′, *GAPDH*(F): 5′GAAGGTGAAGGTCGGAGTC3′, and *GAPDH*(R): 5′GAAGATGGTGATGGGATTTC3′.

### 2.3 Western blotting and immunoprecipitation

The cellular protein was extracted and resolved using sodium dodecyl sulfate polyacrylamide gel electrophoresis. Subsequently, the proteins were transferred onto a polyvinylidene fluoride membrane, blocked with milk, and incubated overnight at 4°C with either anti-TTBK2 or anti-Moesin. The protein bands were visualized using an enhanced chemiluminescence reagent, and band intensities were quantified using Image Pro Plus software.

For co-immunoprecipitation (Co-IP) experiments, transfected cells were lysed, and protein concentrations were determined. Immunoprecipitation was then carried out using anti-IgG and anti-Myc antibodies. Subsequently, western blotting analysis was performed using anti-Myc or anti-Flag antibodies.

### 2.4 Cell transfection

The full-length complementary (c) DNA of human *TTBK2* was synthesized and cloned into the expression vector pEGFP-N1 (Beijing Olinger Biotechnology Co., Ltd). The *TTBK2* ^T3290C^ mutant plasmid was obtained from the same company. Transfection of full-length cDNA or mutant *TTBK2* plasmids into cells was performed using Lipofectamine 3000 (Invitrogen, Carlsbad, CA, USA).

### 2.5 Isolation of MEF cells and induction of cilia formation

The MEFs were isolated from embryos of E10.5 or E12.5 and maintained them as previously described [16]. Subsequently, the isolated MEFs were transfected with wild-type and mutant (T3290C) *TTBK2* plasmids using Lipofectamine 2000. Cilia formation was induced by shifting cells from 10 to 0.5% fetal bovine serum (FBS) and maintaining them under low serum conditions for 48 h.

### 2.6 TTBK2 enzyme activity

TTBK2 enzyme activity was assessed utilizing the Universal Kinase Assay Kit (Abcam, ab138879). Samples were prepared and processed following the manufacturer’s instructions. The procedure for conducting the ADP Assay is as follows: prepare a 50× ADP Sensor I stock solution by combining 50 µL of DMSO with the vial of ADP Sensor I. Subsequently, generate the ADP Sensor by mixing 50 µL of the 50× ADP Sensor I stock solution with the vial of ADP Sensor II. Then, add 20 µL of ADP Sensor Buffer and 10 µL of ADP Sensor to each well containing 20 µL of kinase reaction solution, resulting in a total ADP assay volume of 50 µL/well. Incubate the reaction mixture at room temperature for 30 min, and measure the fluorescence intensity using a fluorescence plate reader set at *E*_x_/*E*_m_ = 540/590 nm (cutoff 570 nm).

### 2.7 Immunofluorescence

The transfected cells were cultured in a 24-well plate with low serum (0.5% FBS) for 48 h, followed by fixation in 4% paraformaldehyde for 30 min. Subsequently, the cells were permeabilized with 0.1% Triton X-100 for 15 min after washing with phosphate-buffered saline. After washing again, the cell was blocked by 5% bovine serum albumin for 2 h at 37°C. Next, the cells were then incubated overnight at 4°C with primary antibodies, ARL13b (66739-1-Ig; Proteintech, 1:200) and γ-tubulin (ab179503; Abcam, 1:500), followed by 1-h incubation at room temperature with secondary antibodies (CY3 R 1:50, FITC M 1:50). Finally, the cells were stained with DAPI in phosphate-buffered solution (PBS) for 10 min, washed with PBS, and fixed on a glass slide.

### 2.8 Statistical analysis

Data were analyzed using GraphPad Prism 7 software and presented as means ± standard deviation. Two groups of data were analyzed by Student’s *t*-test. For multiple comparisons, we used one-way analysis of variance and the Tukey honestly significant difference test. *P  <*  0.05 was considered significant.


**Ethical approval:** The research related to human use has been complied with all the relevant national regulations and institutional policies and in accordance with the tenets of the Helsinki Declaration and has been approved by the authors’ institutional review board or equivalent committee. This study was approved by the Second Affiliated Hospital of Nanchang University.
**Informed consent:** Informed consent has been obtained from all individuals included in this study.

## 3 Results

### 3.1 TTBK2^T3290C^ MUT in SCA11 does not affect its mRNA and protein expression

**Figure 1:**
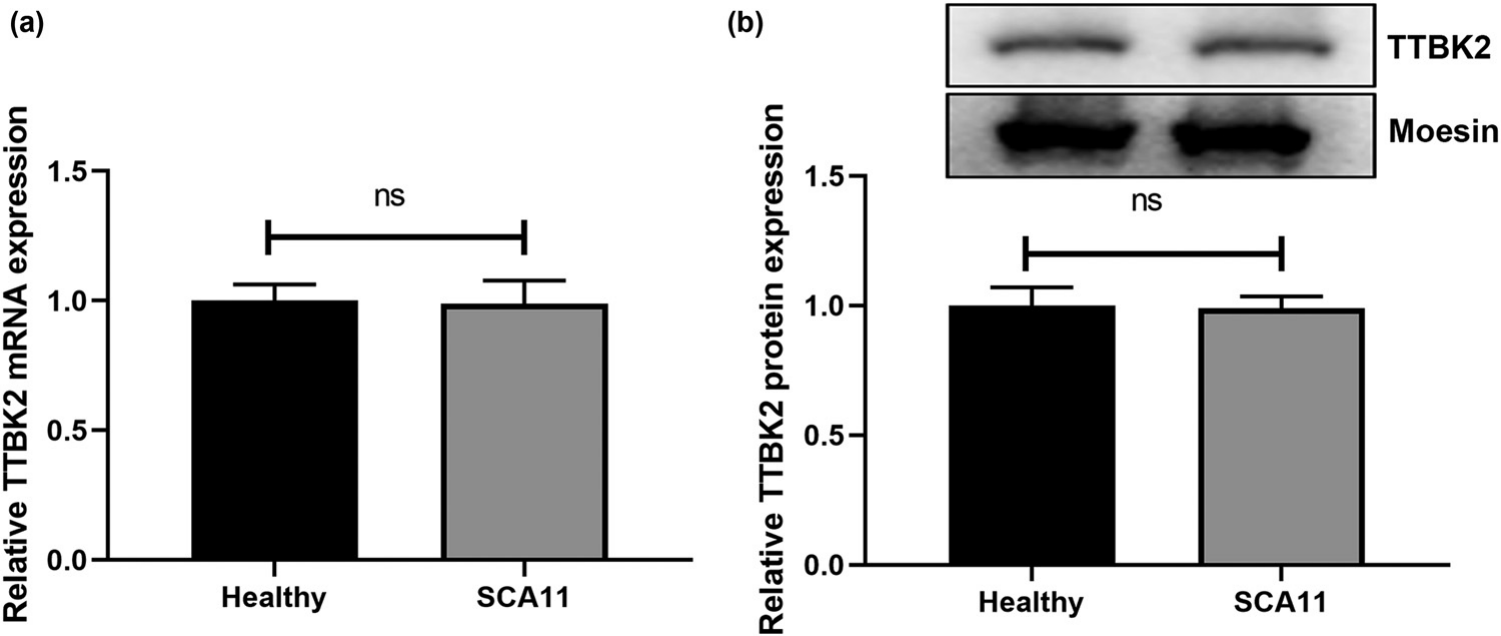
TTBK2 expression in SCA11 patients. Peripheral blood and lymphocytes were extracted from patients (pathogenic variant individuals, mutant types) and healthy individuals (healthy individuals, wild-type) in the SCA11 family. (a) The *TTBK2* mRNA level was detected by RT-PCR. (b) The TTBK2 protein level was detected by western blotting (membrane tissue extension spike protein, Moesin as the control). Results are pooled from three independent experiments. Statistical comparison was performed by Student’s *t*-test. ns indicates no significance.

To compare the expression levels of TTBK2 in individuals with pathogenic variants (mutant type, *n* = 5) and healthy controls (wild type, *n* = 6) within the SCA11 family, peripheral blood samples were collected for lymphocyte extraction. The mRNA and protein expression of TTBK2 were subsequently analyzed using RT-PCR and Western blot techniques. Statistical evaluation revealed no significant difference in *TTBK2* mRNA expression between the patient group and healthy controls (Figure 1a, *p* = 0.8536). Similarly, protein expression analysis showed no significant difference between the two groups (Figure 1b, *p* = 0.8636). These findings suggested that the *TTBK2* ^*T3290C*^ MUT in SCA11 does not impact its gene or protein expression.

### 3.2 The SCA11-associated TTBK2^T3290C^ MUT does not affect its protein expression and enzyme activity

**Figure 2:**
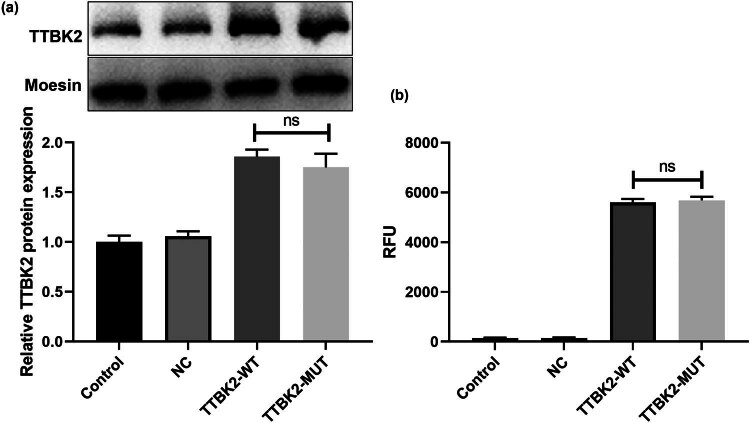
*TTBK2* ^T3290C^ mutation does not affect protein expression and enzyme activity. The empty, wild-type, and mutant (T3290C) *TTBK2* plasmids were transfected into HEK-293 cells, and the untransfected cell as the control group. (a) The TTBK2 protein level was detected by western blotting. (b) The TTBK2 enzyme activity was measured by Universal Kinase Assay Kit. Results are pooled from three independent experiments. Statistical comparison was performed by Student’s *t*-test. ns indicates no significance.

To elucidate the impact of the SCA11-associated *TTBK2* ^T3290C^ MUT on its expression and enzymatic function, we engineered wild-type and mutant (T3290C) *TTBK2* plasmids and transfected them into HEK-293 cells. Non-transfected cells served as controls, while cells transfected with empty plasmids were treated as negative controls (NC). Subsequently, TTBK2 protein levels and kinase activity were assessed. Western blot analysis revealed that the SCA11-associated *TTBK2* ^T3290C^ MUT did not alter its protein expression (Figure 2a, *p* = 0.6544). Data from the Universal Kinase Assay Kit indicated that the *TTBK2* ^T3290C^ MUT did not influence TTBK2’s enzymatic activity (Figure 2b, *p* = 0.9194). These findings suggested that the SCA11-associated *TTBK2* ^T3290C^ MUT does not affect its protein expression or functional activity.

### 3.3 TTBK2^T3290C^ MUT reduces cilia formation in embryonic cells

**Figure 3:**
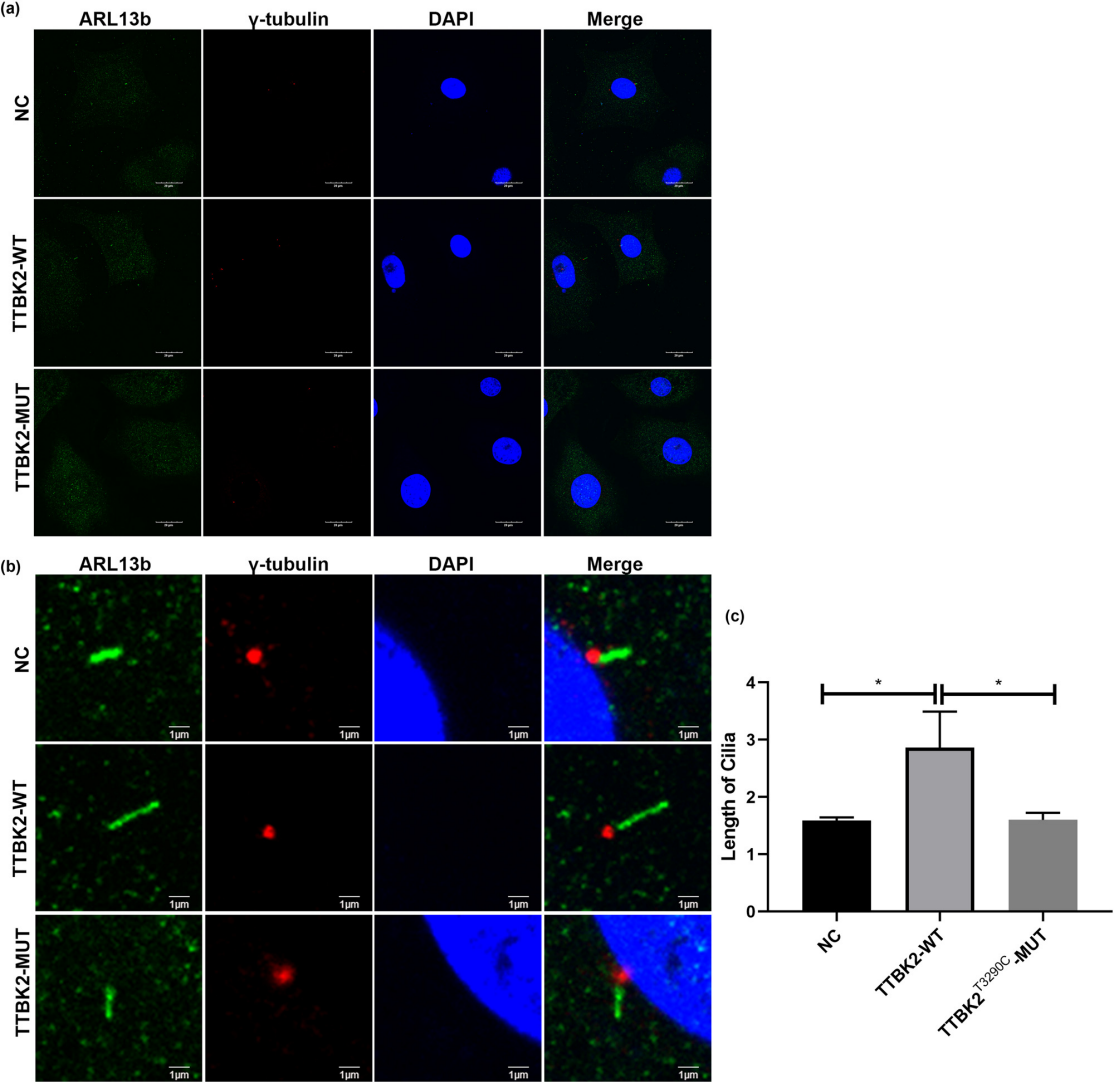
SCA11-associated *TTBK2* ^T3290C^ mutation affects cilia formation in MEF cells. Mouse embryonic MEF cells were isolated and divided into three groups, transfected with empty (NC), wild-type, and mutant (T3290C) *TTBK2* plasmid, respectively. Then, the cells were maintained under low serum conditions for 48 h to induce cilia formation. (a) and (b) Cilia were immunostained for ARL13b (green) to label cilia and γ-Tubulin (red) to label centrosomes, scar bar = 20 and 1 µm. (c) Quantification of the cilia length from MEFs. Results are pooled from three independent experiments. Statistical comparison was performed by Student’s *t*-test. *p < 0.05.

Next, to investigate the effect of SCA11-associated *TTBK2* ^T3290C^ MUT on MEF cell cilia formation, we isolated mouse embryonic MEF cells and transfected them with empty (NC), wild-type, and mutant *TTBK2* plasmids. Immunofluorescence staining for the primary cilia marker ARL13B was utilized to assess cilia formation, with γ-Tubulin serving as a centrosomal marker (Figure 3a and b). Our findings demonstrated that MEF cells transfected with the wild-type *TTBK2* plasmid exhibited a significantly greater ciliary length compared to those transfected with the NC empty plasmid (*p* = 0.0104). Conversely, MEF cells transfected with the *TTBK2* ^T3290C^ mutant plasmid displayed shorter cilia than those transfected with the wild-type *TTBK2* plasmid (*p* = 0.0108) (Figure 3c). These results indicated that the *TTBK2* ^T3290C^ MUT impairs cilia formation in MEF cells.

### 3.4 TTBK2^T3290C^ MUT reduces its binding to Cep164 protein

**Figure 4:**
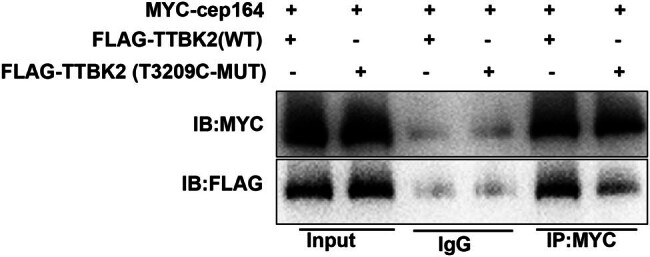
*TTBK2* ^T3290C^ mutation reduces its binding to Cep164 protein. HEK-293 cells were transfected with MYC-Cep164 plasmids and wild-type (Flag-*TTBK2*)/mutant (Flag-*TTBK2* ^T3290C^) plasmid. Pull-down assessment for interaction between TTBK2 and Cep164 *in vitro*.

The aforementioned study demonstrated that the SCA11-associated *TTBK2* ^T3290C^ MUT does not impact TTBK2 protein expression or enzymatic activity, but it significantly impedes ciliogenesis. To elucidate the mechanism by which the SCA11-associated *TTBK2* ^T3290C^ MUT influences ciliogenesis, we assessed the impact of the *TTBK2* ^T3290C^ MUT on its interaction with the Cep164 protein using Co-IP assays. HEK-293 cells were transfected with MYC-Cep164 plasmids alongside either wild-type (Flag-*TTBK2*) or mutant (Flag-*TTBK2* ^T3290C^) plasmids. The findings revealed that the *TTBK2* ^T3290C^ MUT diminished its binding affinity to the Cep164 protein (Figure 4).

## 4 Discussion

SCA11 is a very rare progressive degenerative disease. The diagnosis of SCA11 mainly depends on clinical features and identification of *TTBK2* MUTs. Previously, we documented a Chinese SCA11 pedigree, wherein 10 affected individuals harbored a novel heterozygous *TTBK2* MUT, c.3290T>C (exon 15), which results in an amino acid substitution (p.Val1097Ala) [15]. Although categorized as potentially benign, our prior studies [15] suggested that this variant aligns with the SCA11 diagnosis. Primary cilia exert a crucial regulatory influence on neurogenesis, influencing neuronal differentiation, proliferation, and migration during corticogenesis [17,18,19,20]. Hence, this study aimed to elucidate the impact of the *TTBK2* ^T3290C^ MUT linked to SCA11 on TTBK2 expression, function, and ciliogenesis.

Research indicates that TTBK2 sustains primary cilia by inhibiting their breakdown, thereby fostering the proliferation of granule neural progenitor cells and modulating cerebellar development [21]. Pathological phosphorylation of TDP-43 by TTBK2 may precipitate neurodegeneration [22]. Meanwhile, numerous studies affirmed that TTBK2 is a crucial ciliogenesis regulator [23,24]. Bowie et al.’s [7] study demonstrated that SCA11-associated MUT disrupts the function of full-length *TTBK2* in ciliogenesis mediation. Goetz et al. [25] proved that dominant truncating MUTs in human *TTBK2* cause SCA11, and these mutant proteins do not promote ciliogenesis and inhibit ciliogenesis in wild-type cells. Consistent with the findings of Bowie et al. and Goetz et al., our study also observed that the *TTBK2* ^T3290C^ MUT impeded ciliogenesis. The *TTBK2* MUTs led to either nonsense-mediated decay of the mutant transcripts or the production of truncated TTBK2 proteins with varying lengths due to premature stop codons in the mRNA. The nonsense-mediated decay or the presence of truncated TTBK2 protein significantly diminished kinase activity and acted as a dominant negative allele, interfering with the function of the wild-type TTBK2 protein [3]. As reported by Bowie et al. [7], truncated proteins generated by SCA11-related *TTBK2* MUT can disrupt the function of full-length *TTBK2*, thus affecting ciliogenesis. Our study found no difference in expression of TTBK2 in peripheral blood lymphocytes between patients (pathogenic variant individuals, mutant types) and healthy individuals (healthy individuals, wild type) in the SCA11 family. The *TTBK2* ^T3290C^ MUT did not impact TTBK2 protein expression or enzyme activity, differing from Bouskila et al.’s findings, suggesting that *TTBK2* ^T3290C^ MUT may interfere with cilia formation through alternative mechanisms.

Gene MUTs can affect protein–protein interactions, as evidenced by Liu et al. [26], who reported that the *MPP2* ^N315^ MUT exhibited enhanced binding affinity to ANXA2 compared to *MPP2* ^K315^. Cep164 is localized to the apical domain of the mother centriole, forming a molecular bridge between the mother centriole and the membrane biogenesis machinery essential for cilia initiation [27]. Previous studies identified the recruitment of TTBK2 by the distal appendage protein Cep164 as an early event in ciliogenesis, with Cep164 MUTs disrupting the Cep164-TTBK2 complex [12]. We have confirmed that the *TTBK2* ^T3290C^ MUT diminished its binding affinity to the Cep164 protein, implying that *TTBK2* ^T3290C^ MUT in SCA11 may impair cilia formation by reducing binding to Cep164. TTBK2 interacts with Cep164 via its proline-rich motif. Oda et al. [14] established that Cep164 binding is crucial for TTBK2’s role in promoting ciliogenesis. Further investigations revealed that TTBK2 can phosphorylate Cep164 and Cep97, inhibiting the interaction between Cep164 and Dishevelled-3, a key ciliogenesis regulator, in a kinase activity-dependent manner.

The MUT site (c.3290T>C, p.Val1097Ala, Figure S2) is located outside the protease active region, and our results demonstrated that the *TTBK2* ^T3290C^ MUT did not impact TTBK2 protease activity. However, this MUT decreased TTBK2’s binding to the Cep164 protein. The mechanism by which *TTBK2* ^T3290C^ MUT affects its interaction with Cep164 protein still needs further exploration. We hypothesize that the (c.3290T>C, p.Val1097Ala) MUT may induce secondary and tertiary structural alterations in the protein, thereby influencing TTBK2’s interaction with other proteins such as Cep164. This article evaluates the effects of MUTs on TTBK2 protein expression, enzyme activity, cilia formation, and binding to Cep164 protein through cellular assays. Future research will involve RNA sequencing analysis of wild-type and mutant *TTBK2* in mouse cerebellar neurons to identify distinct functional pathways and elucidate the mechanisms underlying the onset and progression of the *TTBK2* ^T3290C^ MUT.

## 5 Conclusion

In summary, this study corroborates the link between the *TTBK2* ^T3290C^ MUT and SCA11. Our findings indicate no significant difference in TTBK2 expression levels between SCA11 patients and healthy controls. Although the *TTBK2* ^T3290C^ MUT does not alter its protein expression or enzymatic activity, it impairs ciliogenesis via a mechanism involving altered interaction with the Cep164 protein. This research provides new theoretical insights into the pathogenic mechanisms of SCA11.

## Supplementary Material

Supplementary Figure
