# Supplementary material for: TTBK2 T3290C mutation in spinocerebellar ataxia 11 interferes with ciliogenesis
Source: Transl Neurosci. 2024 Oct 3;15(1):20220353. doi: 10.1515/tnsci-2022-0353 (PMC11459611; doi:10.1515/tnsci-2022-0353)
Supplement: Supplementary Figure [file tnsci-2022-0353-sm.pdf]

Supplementary material

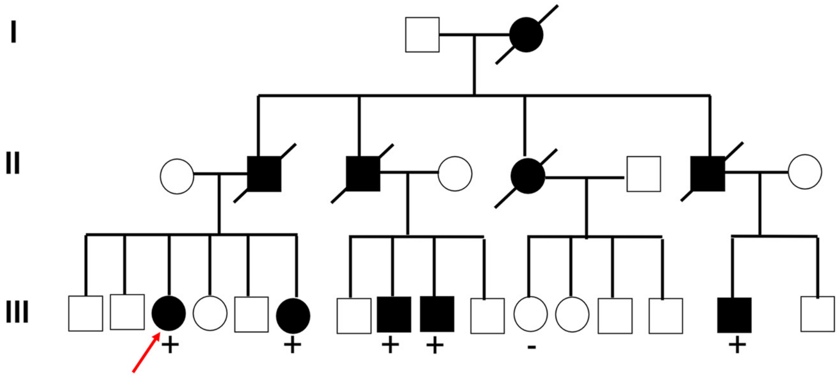

Figure S1: Pedigree of the first Chinese SCA11-affected family. The arrow indicates the patient; circles indicate female and squares indicate male family members; solid symbols indicate affected family members; + and – signs indicate individuals with and without the *TTBK2* mutation, respectively; the/ symbols indicate deceased family members.

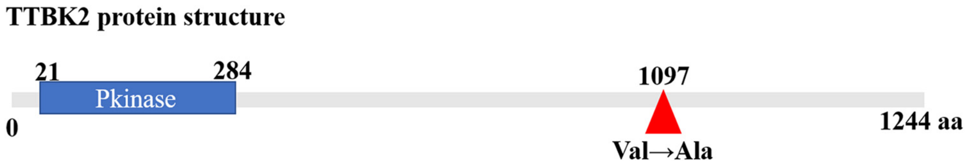

Figure S2: TTBK2 protein structure. TTBK2 has N-terminal kinase domain (amino acids 21-284).
